# Meat production characteristics of Turkish native breeds: II. meat quality, fatty acid, and cholesterol profile of lambs

**DOI:** 10.5194/aab-62-41-2019

**Published:** 2019-02-01

**Authors:** Yüksel Aksoy, Ümran Çiçek, Uğur Şen, Emre Şirin, Mustafa Uğurlu, Alper Önenç, Mehmet Kuran, Zafer Ulutaş

**Affiliations:** 1 Department of Animal Science, Faculty of Agriculture, Osmangazi University, 26160, Eskisehir, Turkey; 2 Department of Animal Science, Faculty of Agriculture, Osmangazi University, 26160, Eskisehir, Turkey; 3 Department of Agricultural Biotechnology, Faculty of Agriculture, Ondokuz Mayis University, 55139, Samsun, Turkey; 4 Department of Agricultural Biotechnology, Faculty of Agriculture, Ahi Evran University, 40100, Kirsehir, Turkey; 5 Department of Animal Science, Faculty of Veterinary Medicine, Ondokuz Mayis University, 55139, Samsun, Turkey; 6 Department of Animal Science, Faculty of Agriculture, Namik Kemal University, 59000, Tekirdag, Turkey; 7 Department of Animal Production and Technologies, Ayhan Sahenk Faculty of Agricultural Sciences and Technologies, Omer Halisdemir University, 51240, Nigde, Turkey

## Abstract

The study conducted a comparison of meat quality, fatty acid profile, and cholesterol amounts of longissimus dorsi (LD) and semitendinosus (ST) muscles of male lambs born to Turkish indigenous sheep breeds raised under intensive conditions. A total of 36 singleton male lambs were used as experimental animals of the Akkaraman (A), Morkaraman (M), Awassi (IW), Karayaka (KR), Kıvırcık (KV), and Middle Anatolian Merino (MAM) pure breeds. All lambs were fed the same diet until they reached a target weight of 40 kg weight. After the feeding period, all lambs were slaughtered and LD and ST muscle samples were collected to determine meat quality traits, fatty acid profile, and cholesterol amounts. Although there were no significant differences between lambs in terms of the fatty acid profile of LD and ST muscles, KR lambs had a higher cholesterol content in both muscles in comparison with the lambs born to other breeds (p<0.05). While water-holding capacity, dripping loss, pH, color, dry matter, ash, and intramuscular fat values of ST muscles showed differences among breeds (p<0.05), dripping loss, pH, cooking loss, color, dry matter, ash, protein, and intramuscular fat values of LD muscles differed between breeds (p<0.05). The data of the current study indicated that meat quality characteristics and cholesterol contents of Turkish indigenous breeds showed differences, and these differences may be used for alternative lamb meat production for the consumer.

## 1 Introduction

Fat content and sensory quality traits such as tenderness, flavor, and color of lamb meat are very important issues when buying or consuming lamb meat (Sirin et al., 2017). Moreover, previous studies reported that breed or genotype is an important factor influencing lamb meat quality (Santos-Silva et al., 2002; Martinez-Cerezo et al., 2005; Sirin et al., 2017). The raising of sheep flocks for meat production is carried out with native breeds in many countries. Therefore, it is possible to offer meat with different properties such as different fat contents by the determination of the quality and nutrient content of the meat obtained from native sheep breeds. There are more than 32 million sheep with various native breeds in different geographical regions of Turkey (TurkStat, 2016). Akkaraman (A), Morkaraman (M), and Awassi (IW), which are classified as fat-tail breeds, and Karayaka (KR), Kıvırcık (KV), and Middle Anatolian Merino (MAM), which are classified as thin-tail breeds, are commonly raised indigenous sheep breeds, which are well suited to a variety of harsh geographic and climatic conditions. Thus, they constitute approximately 80 % of the sheep population in Turkey (TurkStat, 2016). Male lambs of these breeds are used as fattening material for meat production, and they constitute an important source of red meat production under harsh climate conditions (Sirin et al., 2017).

Our previous study (Aksoy et al., 2018) showed that there were significant differences among male lambs of A, M, IW, KR, KV, and MAM Turkish indigenous breeds in terms of slaughter and carcass traits. Moreover, many studies were conducted on determining the fattening performance and carcass characteristics of A, M, IW, KV, KR, and MAM sheep breeds (Macit, 2002; Ekiz et al., 2009; Yilmaz et al., 2009; Esenbuga et al., 2009; Sen et al., 2011). However, there is a lack of studies that compare the meat quality, fatty acid, and cholesterol profile of these breeds under the same intensive conditions.

The aim of the current study was therefore to determine comparatively some meat quality parameters, fatty acid, and the cholesterol profile of longissimus dorsi (LD) and semitendinosus (ST) muscles of A, M, IW, KV, KR, and MAM lambs, which were raised under the same intensive conditions.

## 2 Materials and methods

The experimental procedures were approved by the Local Animal Care and Ethics Committee of Gaziosmanpasa University, Tokat, Turkey, ensuring compliance with EC Directive 86/609/EEC for animal experiments. The experiment was conducted at the Agricultural Research and Application Farm of Gaziosmanpasa University, Tokat, Turkey (40${}^{\circ }$31${}^{\prime }$ N, 36${}^{\circ }$53${}^{\prime }$ E; 650 m a.s.l.). A total of 36 singleton male lambs of Akkaraman (n=6), Morkaraman (n=6), Awassi (n=6), Karayaka (n=6), Kıvırcık (n=6), and Middle Anatolian Merino (n=6) sheep breeds were used as experimental animals. They were randomly selected at weaning age after 90 days with an average body weight of 20 kg. After weaning, all lambs were subject to a fattening period, and they were then slaughtered when they reached a live weight of 40 kg. Before the fattening period, all lambs were allowed to adapt to the nutritional treatments for 1 week. The lambs were fed ad libitum with concentrate feed and approximately 100 g day${}^{-1}$ of alfalfa hay. All lambs had free access to water and a mineral stone during the fattening period. The nutrient contents of the feeds used during the fattening period are shown in Table 1.

**Table 1:** The chemical composition of concentrate feed and alfalfa hay.

Nutrient content	Concentrated	Alfalfa hay
Dry matter (%)	93.10	94.00
Crude protein (%)	15.20	15.00
Crude oil (%)	2.23	0.74
Crude ash	8.60	10.30
ADF	29.41	59.75
NDF	30.22	58.22
Metabolic energy (kcal kg${}^{-1}$)	2690.00	1878.00

ADF: acid detergent fiber; NDF: neutral detergent fiber.

None of the lambs were fed overnight (approximately 16 h) before the slaughter process. Lambs were weighed to determine the slaughter weight, and they were slaughtered in standard commercial slaughter procedures. LD and ST muscles samples were used for the determination of meat quality parameters, fatty acid composition, and cholesterol content due to there being well-known correlations between the LD and ST muscle characteristics with carcass sections (Sen et al., 2011; Yarali et al., 2014). Following slaughter, the carcasses of all lambs were chilled for 24 h at 4 ${}^{\circ }$C. After chilling, approximately 150–200 g muscle samples were collected from the central parts of the midsection of the whole LD and ST muscles taken from the left side of the carcasses to determine the meat quality traits. After homogenizing the meat sample, dry matter, protein (N × 6.25), intramuscular fat, and ash contents of LD and ST muscles were analyzed according to the AOAC (1990) procedures. The water-holding capacity of LD and ST muscle samples (approximately 25 g) were determined by the filter-paper press method (Van Oeckel et al., 1999) with some modifications. Approximately 50 g LD and ST muscle samples were vacuum-packed and stored at $-20{}^{\circ }$ for 1 week to evaluate thawing loss values (Rahman et al., 2014). The sample packages were thawed under tap water, and then the thawing loss values were expressed as a percent of water (Rahman et al., 2014). To determine the dripping loss values of LD and ST muscle samples, approximately 50 g of each muscle were vacuum-packaged and stored at 4 ${}^{\circ }$C for 7 days. The dripping loss values (%) were measured on the third and seventh day of storage (Sen et al., 2011). Cooking loss values of LD and ST muscle samples were determined according to Sen et al. (2011). The muscle samples were put in plastic bags and cooked for 40 min in a water bath at 70 ${}^{\circ }$C. Following the cooking step the samples were cooled under tap water. The cooking loss values were calculated as percentage of weight loss. The pH value of muscle samples was measured at 24 h after slaughter by using a pH meter with a puncture electrode (Testo 205, Lenzkirch, Germany). CIE (International Commission on lumination) lightness ($L^{*}$), redness ($a^{*}$), and yellowness ($b^{*}$) value measurements were taken by using a chronometer (Konica Minolta CR-300, Minolta Co., Ltd., Osaka, Japan) at 24 h after slaughter. The protein, ash, and intramuscular fat content was determined as a percentage of dry (samples were kept for 12 h at 105 ${}^{\circ }$C) muscle sample weight (Sen et al., 2011). Water-holding capacity, drip loss, cooking loss, and frozen–thawing loss were determined as a percentage of fresh muscle sample weight (Sen et al., 2011). The lipid extraction from muscle samples for the determination of fatty acid composition and cholesterol amounts was performed with chloroform/methanol (2:1), as described by Folch et al. (1957). The fatty acid composition and cholesterol content of the muscle samples were analyzed as described by Aksoy and Ulutas (2016). The fatty acid compositions were expressed in percentage of methyl esters. The cholesterol amounts of muscle samples were determined as mg cholesterol 100 g${}^{-1}$ sample.

The statistical analysis was conducted on a completely randomized design for traits. The statistical analyses were performed using SPSS statistical software (1999). Significant differences between means were tested by Duncan's multiple comparison tests. Results were computed as mean ± SE, and statistical significance was determined at the level of p<0.05.

**Table 2:** Meat quality parameters and chemical composition of longissimus dorsi muscle from male lambs born to Turkish indigenous sheep breeds.

Traits	A	M	KR	KV	IW	MAM
pH	5.62 ± 0.03${}^{\text{c}}$	5.71 ± 0.02${}^{\text{b}}$	5.69 ± 0.02${}^{\text{bc}}$	5.72 ± 0.02${}^{\text{ab}}$	5.79 ± 0.03${}^{\text{a}}$	5.61 ± 0.02${}^{\text{c}}$
Water-holding capacity	34.15 ± 0.67	35.23 ± 0.69	34.23 ± 0.61	33.02 ± 0.63	32.40 ± 0.67	34.03 ± 0.86
Drip loss (%)						
Three days	8.73 ± 0.58${}^{\text{ab}}$	10.39 ± 0.72${}^{\text{a}}$	10.13 ± 0.62${}^{\text{ab}}$	9.72 ± 0.58${}^{\text{ab}}$	8.08 ± 0.62${}^{\text{b}}$	9.34 ± 0.58${}^{\text{ab}}$
Seven days	10.95 ± 0.60${}^{\text{bc}}$	14.87 ± 1.02${}^{\text{a}}$	13.00 ± 0.62${}^{\text{ab}}$	12.23 ± 0.60${}^{\text{bc}}$	10.38 ± 0.66${}^{\text{c}}$	11.35 ± 0.69${}^{\text{bc}}$
Frozen–thawing loss (%)	13.31 ± 0.98	13.31 ± 1.12	11.64 ± 0.89	10.62 ± 0.89	13.80 ± 0.93	13.94 ± 0.93
Cooking loss (%)	28.48 ± 0.60${}^{\text{bc}}$	30.54 ± 0.63${}^{\text{a}}$	30.20 ± 0.60${}^{\text{ab}}$	28.48 ± 0.58${}^{\text{bc}}$	29.52 ± 0.67${}^{\text{abc}}$	27.73 ± 0.55${}^{\text{c}}$
Color						
$L^{*}$	46.59 ± 0.53${}^{\text{a}}$	44.48 ± 0.47${}^{\text{b}}$	42.98 ± 0.43${}^{\text{c}}$	44.08 ± 0.47${}^{\text{b}}$	41.83 ± 0.47${}^{\text{c}}$	42.28 ± 0.53${}^{\text{c}}$
$a^{*}$	18.09 ± 0.41${}^{\text{c}}$	19.02 ± 0.36${}^{\text{bc}}$	20.18 ± 0.32${}^{\text{a}}$	19.53 ± 0.36${}^{\text{ab}}$	17.83 ± 0.36${}^{\text{c}}$	19.60 ± 0.41${}^{\text{ab}}$
$b^{*}$	5.48 ± 0.36${}^{\text{bc}}$	6.42 ± 0.32${}^{\text{ab}}$	6.53 ± 0.29${}^{\text{a}}$	6.29 ± 0.32${}^{\text{ab}}$	5.26 ± 0.32${}^{\text{c}}$	5.17 ± 0.36${}^{\text{c}}$
Dry matter (%)	23.09 ± 0.26${}^{\text{b}}$	23.41 ± 0.25${}^{\text{b}}$	24.75 ± 0.25${}^{\text{a}}$	24.77 ± 0.25${}^{\text{a}}$	23.31 ± 0.36${}^{\text{b}}$	24.32 ± 0.31${}^{\text{a}}$
Ash	1.07 ± 0.01${}^{\text{b}}$	1.07 ± 0.01${}^{\text{b}}$	1.06 ± 0.01${}^{\text{b}}$	1.08 ± 0.01${}^{\text{b}}$	1.11 ± 0.01${}^{\text{a}}$	1.08 ± 0.01${}^{\text{b}}$
Protein	19.72 ± 0.26${}^{\text{abc}}$	19.71 ± 0.26${}^{\text{abc}}$	18.98 ± 0.31${}^{\text{c}}$	19.38 ± 0.25${}^{\text{bc}}$	20.49 ± 0.44${}^{\text{a}}$	20.31 ± 0.31${}^{\text{ab}}$
Intramuscular fat	1.62 ± 0.14${}^{\text{b}}$	1.98 ± 0.14${}^{\text{b}}$	3.72 ± 0.16${}^{\text{a}}$	3.62 ± 0.13${}^{\text{a}}$	2.04 ± 0.23${}^{\text{b}}$	2.19 ± 0.16${}^{\text{b}}$

${}^{a,b,c}$: The differences indicated by different letters on the same line are significant. $L^{*}$: lightness; $a^{*}$: redness; $b^{*}$: yellowness. A: Akkaraman; M: Morkaraman; IW: Awassi; KR: Karayaka; KV: Kıvırcık; MAM: Middle Anatolian Merino.

## 3 Results

The meat quality parameters and chemical composition of LD muscles of male lambs born to Turkish indigenous sheep breeds are given in Table 2. Significant variation was detected in the pH values of LD muscles among the breeds (p<0.05). The LD muscle pH value of IW lambs was relatively higher (p<0.05) than those of other breeds, except for KV lambs. There were significant differences among lambs born to A, M, IW, KR, KV, and MAM breeds in terms of dripping loss and cooking loos values (p<0.05). M lambs had relatively higher dripping loss values on day 3 and day 7 when compared to other breeds (p<0.05). Similarly, the cooking loss value of M lambs was higher than those of other breeds (p<0.05). Although, dripping loss and cooking loss values showed differences among breeds, breed had no significant effect on water-holding capacity and thawing loss values (p>0.05). In the present study, the differences among lambs born to A, M, IW, KR, KV, and MAM Turkish pure breeds in terms of $L^{*}$, $a^{*}$, and $b^{*}$ values were significant (p<0.05). Although the highest $L^{*}$ value was determined in A lambs, KR lambs had higher $a^{*}$ and $b^{*}$ values. There were significant differences among lambs born to different Turkish pure breeds in terms of the chemical composition of the LD muscle (p<0.05). KR, KV, and MAM lambs had a higher percentage of dry matter than those of the A, M, and IW breeds (p<0.05). It was seen that KR lambs had the highest percentage of intramuscular fat (except for KV lambs) in comparison with the other breeds (p<0.05). Total protein and ash values of IW lambs were higher than lambs born to different Turkish pure breeds (p<0.05).

**Table 3:** Meat quality parameters and chemical composition of the semitendinosus muscle from male lambs born to Turkish indigenous sheep breeds.

Traits	A	M	KR	KV	IW	MAM
pH	5.60 ± 0.01${}^{d}$	5.73 ± 0.01${}^{a}$	5.68 ± 0.01${}^{\mathrm{ab}}$	5.61 ± 0.01${}^{\mathrm{cd}}$	5.66 ± 0.02${}^{\mathrm{bc}}$	5.65 ± 0.01${}^{\mathrm{bcd}}$
Water-holding capacity	33.49 ± 0.63${}^{\mathrm{ab}}$	35.42 ± 0.66${}^{a}$	34.04 ± 0.58${}^{\mathrm{ab}}$	33.11 ± 0.58${}^{b}$	32.59 ± 0.63${}^{b}$	33.01 ± 0.82${}^{b}$
Drip loss (%)						
Three days	8.57 ± 0.66${}^{b}$	9.27 ± 0.70${}^{\mathrm{ab}}$	10.52 ± 0.73${}^{a}$	8.52 ± 0.64${}^{b}$	9.25 ± 0.70${}^{\mathrm{ab}}$	8.05 ± 0.64${}^{b}$
Seven days	10.26 ± 0.84${}^{\mathrm{bc}}$	13.31 ± 1.00${}^{a}$	13.35 ± 0.89${}^{a}$	9.36 ± 0.82${}^{c}$	12.56 ± 0.89${}^{\mathrm{ab}}$	10.84 ± 0.82${}^{\mathrm{abc}}$
Frozen–thawing loss (%)	11.26 ± 0.89	10.98 ± 1.00	10.89 ± 0.89	12.81 ± 0.85	13.22 ± 0.89	9.46 ± 0.89
Cooking loss (%)	31.32 ± 0.59	31.84 ± 0.69	30.89 ± 0.65	29.56 ± 0.59	30.67 ± 0.69	30.23 ± 0.62
Color						
$L^{*}$	47.08 ± 0.69${}^{a}$	42.69 ± 0.57${}^{\mathrm{bc}}$	41.28 ± 0.50${}^{c}$	42.01 ± 0.56${}^{\mathrm{bc}}$	42.16 ± 0.58${}^{\mathrm{bc}}$	43.52 ± 0.66${}^{b}$
$a^{*}$	17.95 ± 0.41${}^{c}$	19.93 ± 0.34${}^{b}$	21.10 ± 0.30${}^{a}$	20.77 ± 0.34${}^{\mathrm{ab}}$	20.87 ± 0.35${}^{\mathrm{ab}}$	20.13 ± 0.40${}^{\mathrm{ab}}$
$b^{*}$	5.80 ± 0.31${}^{c}$	6.53 ± 0.25${}^{b}$	7.51 ± 0.22${}^{a}$	7.37 ± 0.25${}^{a}$	7.29 ± 0.26${}^{a}$	6.93 ± 0.29${}^{\mathrm{ab}}$
Dry matter (%)	22.92 ± 0.31${}^{c}$	23.06 ± 0.31${}^{\mathrm{bc}}$	24.00 ± 0.31${}^{\mathrm{ab}}$	24.72 ± 0.33${}^{a}$	23.44 ± 0.30${}^{\mathrm{bc}}$	24.55 ± 0.33${}^{a}$
Ash	1.13 ± 0.01${}^{a}$	1.07 ± 0.01${}^{c}$	1.09 ± 0.01${}^{\mathrm{bc}}$	1.10 ± 0.01${}^{\mathrm{abc}}$	1.07 ± 0.01${}^{\mathrm{bc}}$	1.11 ± 0.01${}^{\mathrm{ab}}$
Protein	19.98 ± 0.39	19.31 ± 0.39	19.66 ± 0.44	20.37 ± 0.41	20.80 ± 0.47	20.92 ± 0.44
Intramuscular fat	1.15 ± 0.15${}^{c}$	2.01 ± 0.15${}^{b}$	2.70 ± 0.17${}^{a}$	2.52 ± 0.16${}^{a}$	1.37 ± 0.18${}^{c}$	1.85 ± 0.17${}^{b}$

${}^{a,b,c}$: the differences indicated by different letters on the same line are significant. $L^{*}$: lightness; $a^{*}$: redness; $b^{*}$: yellowness. A: Akkaraman; M: Morkaraman; IW: Awassi; KR: Karayaka; KV: Kıvırcık; MAM: Middle Anatolian Merino.

Chemical composition and meat quality parameters of the ST muscle of male lambs born to Turkish indigenous sheep breeds were also determined (Table 3). The pH values of ST muscle samples varied among the lambs born to Turkish pure breeds (p<0.05). ST muscle pH values of M lambs were relatively higher than those of other breeds, except for KV lambs (p<0.05). Significant variation was detected among lambs born to A, M, IW, KR, KV, and MAM Turkish pure breeds in terms of dripping loss and water-holding capacity (p<0.05). KR lambs had the highest dripping loss values measured on both the third and seventh day (p<0.05). The water-holding capacity value of M lambs was relatively higher than those of other breeds (p<0.05). Although dripping loss and water-holding capacity values showed differences among breeds, breed had no significant effect on either cooking loss and thawing loss values. CIE $L^{*}$, $a^{*}$, and $b^{*}$ values showed differences among lamb breeds (p<0.05). Although A lambs had the highest $L^{*}$ value, the lowest $a^{*}$ and $b^{*}$ values were obtained from the same breed (p<0.05). The differences between the chemical compositions of the ST muscle of lambs born to different Turkish pure breeds were statistically significant (p<0.05). Thus, KR, KV, and MAM lambs had a higher percentage of dry matter than those of the A, M, and IW breeds (p<0.05). KR lambs had the highest intramuscular fat (p<0.05) compared to lambs born to other breeds. Although MAM lambs had the highest total protein content, the differences between the breeds were not significant (p>0.05). The ash content of lamb breeds was in the range of 1.07 (IW, M)-1.13 (A) % (p<0.05).

**Table 4:** Fatty acid composition of longissimus dorsi and semitendinosus muscles from male lambs born to Turkish indigenous sheep breeds.

	A	M	KR	KV	IW	MAM
LD						
ΣSFA	31.04 ± 4.57	33.88 ± 4.57	31.67 ± 3.73	33.38 ± 3.73	33.04 ± 4.57	30.95 ± 3.73
ΣMUFA	34.24 ± 4.60	41.65 ± 4.60	36.18 ± 3.76	34.70 ± 3.76	37.88 ± 4.60	31.67 ± 3.76
ΣPUFA 34.72 ± 8.41	24.46 ± 8.41	32.14 ± 6.87	31.91 ± 6.87	29.08 ± 8.41	37.70 ± 6.87	
(ΣMUFA + ΣPUFA) / ΣSFA	2.22 ± 0.42	2.00 ± 0.42	2.16 ± 0.34	2.24 ± 0.34	2.05 ± 0.42	2.26 ± 0.34
Σn-6	22.47 ± 4.09	19.57 ± 4.09	20.29 ± 3.34	14.85 ± 3.34	20.74 ± 4.09	25.84 ± 3.34
Σn-3	5.59 ± 5.52	2.90 ± 1.52	6.82 ± 4.51	13.83 ± 4.51	3.23 ± 5.52	6.30 ± 4.51
Σn-6 / Σn-3	4.36 ± 1.59	6.74 ± 1.59	3.31 ± 1.30	1.80 ± 1.30	6.34 ± 1.59	6.39 ± 1.30
ST						
ΣSFA	28.35 ± 5.82	40.93 ± 5.82	35.61 ± 5.82	31.03 ± 5.82	31.67 ± 8.23	29.44 ± 5.82
ΣMUFA	29.41 ± 3.40	24.22 ± 3.40	44.81 ± 3.40	34.07 ± 3.40	39.84 ± 4.81	34.07 ± 3.40
ΣPUFA	42.39 ± 4.20	34.84 ± 4.20	19.57 ± 4.20	35.88 ± 4.20	28.49 ± 5.94	36.48 ± 4.20
(ΣMUFA+ΣPUFA) / ΣSFA	2.53 ± 0.39	1.71 ± 0.39	1.80 ± 0.39	2.25 ± 0.39	2.15 ± 0.55	2.40 ± 0.39
Σn-6	20.01 ± 2.00	25.59 ± 2.00	13.79 ± 2.00	22.53 ± 2.00	20.91 ± 2.83	25.52 ± 2.00
Σn-3	5.16 ± 0.88	3.57 ± 0.88	1.83 ± 0.88	3.14 ± 0.88	3.51 ± 1.25	3.85 ± 0.88
Σn-6 / Σn-3	4.11 ± 2.10	8.49 ± 2.10	7.76 ± 2.10	7.25 ± 2.10	5.95 ± 2.97	7.06 ± 2.10

LD: longissimus dorsi muscle; ST: semitendinosus muscle. ΣSFA: total saturated fatty acids; ΣMUFA: total monounsaturated fatty acids; ΣPUFA: total polyunsaturated fatty acids; Σn-6: total omega-6 fatty acids; Σn-6: total omega-3 fatty acids. A: Akkaraman; M: Morkaraman; IW: Awassi; KR: Karayaka; KV: Kıvırcık; MAM: Middle Anatolian Merino.

Fatty acid compositions of the longissimus dorsi and semitendinosus muscles of male lambs born to Turkish indigenous sheep breeds are presented in Table 4. There were no significant differences among lambs born to A, M, IW, KR, KV, and MAM Turkish pure breeds in terms of total saturated fatty acids (SFAs), total monounsaturated fatty acids (MUFAs), total polyunsaturated fatty acids (PUFAs), the ratio of total unsaturated fatty acids to total saturated fatty acids, total omega-6 fatty acids (Σn-6), total omega-3 fatty acids (Σn-3), and the ratio of total omega-6 fatty acids to total omega-6 fatty acids (Σn-6 / Σn-3) amounts in either LD or ST muscles (p>0.05). Although the LD muscle of the MAM breed had the highest total PUFA amount (37.70 %), the ST muscle of the A breed had the highest total PUFA amount. The Σn-3 and Σn-6 amounts of LD muscles of all breeds were in the range of 3.23 %–13.83 % and 14.85 %–25.84 %, respectively. It was seen that the Σn-3 and Σn-6 amounts of ST muscles of all breeds were in the range of 1.83 %–5.16 % and 13.79 %–25.52 %, respectively. The results showed that both LD and ST muscles of MAM breed had the highest Σn-6 amounts. The highest measured value was 6.74 in the LD muscle of the M breed, while the LD muscle of the KV breed had the lowest Σn-6 / Σn-3 ratio (1.80). For ST muscles of all breeds, it was determined that the M breed had the highest Σn-6 / Σn-3 ratio (8.49), and the A breed had the lowest Σn-6 / Σn-3 ratio (4.11).

**Figure 1:**
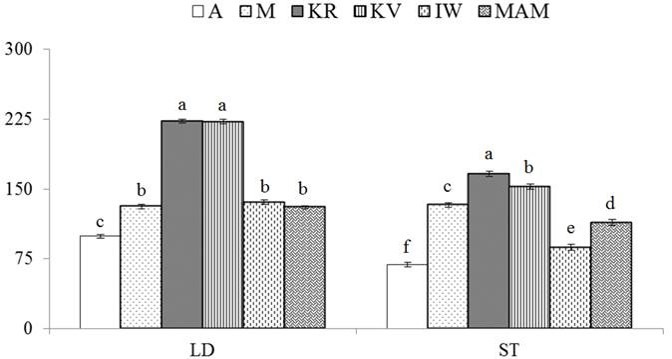
Cholesterol content in longissimus dorsi (LD) and semitendinosus (ST) muscles from male lambs born to Turkish indigenous sheep breeds. A: Akkaraman; M: Morkaraman; IW: Awassi; KR: Karayaka; KV: Kıvırcık; MAM: Middle Anatolian Merino. The error bars represent the standard error of the mean, and bars with different letters are significantly different at p<0.05.

Cholesterol contents of LD and ST muscles of male lambs born to Turkish indigenous sheep breeds were also determined (Fig. 1). The results showed that the differences between the cholesterol contents of lamb breeds were statistically significant for both LD and ST muscles (p<0.05). The total cholesterol contents of LD and ST muscles of all breeds were in the ranges of 99.4–223.28 mg 100 g${}^{-1}$ fat and 68.7–166.2 mg 100 g${}^{-1}$ fat, respectively. The results indicated that both the LD and the ST muscles of the KR breed had the highest total cholesterol contents (p<0.05). The lowest total cholesterol contents were measured as 99.4 mg 100 g${}^{-1}$ fat and 68.7 mg 100 g${}^{-1}$ fat in LD and ST muscles of the A breed, respectively (p<0.05).

## 4 Discussion

The pH value of fresh meat after the rigor mortis phase has important effects on some meat quality characteristics including water-holding capacity and texture. Therefore, the determination of the pH value plays a pivotal role in the assessment of meat quality and consumer preference. Martinez-Cerezo et al. (2005) reported that male lambs of some native Spanish sheep breeds (Rasa Aragonesa, Churra and Spanish Merino) had similar 24 h post mortem pH values. Similarly, Sañudo et al. (1997) reported that the meat pH values of suckling lambs born to Churra, Castellana, Manchega, and Awassi cross breeds were similar. However, some previous studies showed that there were significant differences in the ultimate meat pH among sheep breeds (Hoffman et al., 2003; Hopkins and Fogarty, 1998; Sañudo et al., 2003). The variations in the ultimate meat pH among breeds were generally explained by the metabolic characteristics of muscle fibers in skeletal muscle mass such as glycolytic, oxidative, and oxido-glycolytic activity (Hopkins and Fogarty, 1998; Sen et al., 2016; Sirin et al., 2017) or by preslaughter manipulations (Santos et al., 2007; Ekiz et al., 2009). In the present study, all lambs were subject to similar preslaughter conditions. Additionally, our previous study by Sirin et al. (2017) reported that there were no significant correlations between pH and muscle fiber characteristics. Differences in pH values of LD and ST muscles among lambs obtained from Turkish indigenous sheep breeds may have resulted from the unique structure of the breeds. Devine et al. (1993) reported that the sensory tenderness score of lamb decreases with ultimate pH values between 5.8 and 6.0. In the present study, mean 24 h post mortem pH values of LD and ST muscles of lambs born to Turkish sheep breeds were lower than 5.8 and within the acceptable range.

Water-holding capacity, dripping loss, and cooking loss values are mainly physical meat quality traits and effective in the productivity and quality of meat products. Water-holding capacity, dripping loss, and cooking loss values are related to postmortem biochemical facts such as proteolysis, shrinkage of muscle proteins (actin and myosin), and the destruction of cell walls. These biochemical acts are effective for the release of intercellular water. In addition, the high glycolytic metabolism in muscle results in increases in water loss (i.e., high dripping loss, low water-holding capacity) of meat. The loss of water in meat has an adverse effect on meat quality properties such as tenderness and juiciness. In this study, the highest water loss was determined from the LD muscle of the M breed. This result can be attributed to the type of fiber in muscle. Thus, our previous study reported that type IIB muscle fiber, which has fast glycolytic activity in the LD muscle of the M breed, was more common than in other breeds (Sirin et al., 2017).

The cooking loss values of different indigenous sheep breeds in Turkey ranged between 25.57 % and 34.78 % (Ekiz et al., 2009; Esenbuga et al., 2009; Yakan and Ünal 2010; Ugurlu et al., 2017). In this study, cooking loss values of A, M, KR, KV, IW, and MAM were measured as 31.32 %, 31.84 %, 30.84 %, 29.56 %, 30.17 %, and 30.23 %, respectively. These results indicated that meat lambs of indigenous breeds may not be a disadvantage in terms of marketing.

The color of meat is of the utmost importance for the consumer's impression of the freshness of the product. Thus, consumers generally prefer light red or pink colored lamb meat (Ekiz et al., 2009; Aksoy and Ulutas, 2016). In the present study, the color parameters $L^{*},a^{*}$, and $b^{*}$ were measured over the cold carcasses (24 h after slaughter) in LD and ST muscles, and significant differences were observed between lambs born to Turkish sheep breeds. For lamb meat, acceptable threshold values for $L^{*}$ and $a^{*}$ were reported to be 34–35 and below 19, respectively (Hopkins, 1996). Other studies regarding purchasing decisions by customers for lamb meat reported that the mean $L^{*}$ and $a^{*}$ value is equal to or exceeds 34 and 9.50 respectively; thus, the customer will consider the meat color acceptable (Khliji et al., 2010). Also, under intensive fattening conditions, $L^{*},a^{*}$, and $b^{*}$ values of sheep breeds in Turkey measured at 24 h of postmortem were in the ranges of 37.91–42.72, 16.08–21.26, and 5.60–8.45, respectively (Ekiz et al., 2009; Esenbuga et al., 2009; Yakan and Ünal, 2010; Ugurlu et al., 2017). In the present study, $L^{*},a^{*}$, and $b^{*}$ values of 24 h post mortem were in the ranges of 41.83–47.08, 17.83–21.10, and 5.17–7.51, respectively. The $L^{*}$ value was higher than the acceptable average, while $a^{*}$ was near the acceptable threshold value.

The chemical composition of meat is one of the best predictors of carcass meat composition (Fozooni and Zamiri, 2007). Generally, the chemical composition of lamb meat was determined to be approximately 75 % water and 25 % dry matter. The component of the dry matter is 20 % protein, 3 %–5 % intramuscular fat, 1 % carbohydrates, and 1 % vitamin/mineral (Arnarson, 2015). In the present study, the dry matter (22.92 %–24.75 %), protein (18.98 %–20.92 %), intramuscular fat (1.15 %–3.72 %), and ash (1.07 %–1.13 %) contents of LD and ST muscle samples of male lambs born to Turkish indigenous sheep breeds were considered as acceptable for fresh lamb meat on sale. Thin-tail breeds have a higher fat content in carcasses compared to fat-tail breeds, which store most of the fat in their tails. Previous studies reported that male lambs of the KR sheep breed, which is a thin-tail breed, have tasty meat due to their mosaic distribution pattern of fat among muscle fibers (Ulutas et al., 2010; Sen et al., 2011). At the end of the fattening period in the present study, KR lambs had a higher intramuscular fat content than other breeds. These observations are in agreement with the argument of Sen et al. (2011).

SFA, MUFA, PUFA, Σn-3, and Σn-6 contents are mainly used for determining the nutritional value of the intramuscular fat of meat. Furthermore, their effects on human health have also been related to the ratio of polyunsaturated and saturated fatty acids and the ratio of Σn-6 and Σn-3 fatty acids (Department of Health, 1994). According to the recommendations in Department of Health (1994), the ratios of Σn-6 / Σn-3 and ΣPUFA / ΣSFA should be equal to or below 4.0 and 0.45, respectively. The data obtained in this study for Σn-6 / Σn-3, except for the LD muscle of KR and KV, were above this recommendation. Some studies reported that increasing the amount of concentrate feed in the diet resulted in significant increases in the Σn-6 / Σn-3 ratio of meat (Santos-Silva et al., 2002; Demirel et al., 2006). Therefore, a high ratio of Σn-6 / Σn-3 in this study can be attributed to the amount of concentrate feed in the diet.

Cholesterol is a substance in the natural sterol that is found in the cell membranes of the body tissues of animals and carried in the blood plasma. Cholesterol is found especially in animal products. A small portion of the cholesterol in the body is foodborne; most is synthesized by the body. The synthesis of cholesterol in the body increases due to the body taking in too many saturated fatty acids, the degradation of the fat by the oxidative path, and the stimulation of fat reaction product (McNamara, 2000). However, the World Health Organization maintains its awareness to cholesterol taken with the diet and recommends up to 300 mg day${}^{-1}$ of cholesterol to be taken with the diet (Jimenez-Colmenero et al., 2001). A study on the various hair and wool sheep and their crosses reported that genotype had an effect on cholesterol levels ranging from 50 to 250 mg 100 g${}^{-1}$ fresh meat (Bunch et al., 2004). Another study reported that cholesterol contents of the LD muscle were 63.0 and 63.3 mg 100 g${}^{-1}$ fresh meat for Île de France × Pagliarola and Gentile Di Puglia × Sopravissana while corresponding values for semimembranosus muscle were 75.3 and 67.1 mg 100 g${}^{-1}$ fresh meat, respectively (Salvatori et al., 2004). Previous studies reported that breed is the most important factor regarding the cholesterol content of lamb meat, independently of slaughter weight (Matthes et al., 1998; Nurnberg et al., 1998; Arsenos et al., 2000). The result of the present study showed that genotype, as well as the type of muscle (LD and ST), can markedly influence cholesterol levels. However, the average cholesterol amounts of all indigenous breed were considered acceptable.

As a conclusion, the findings show that sheep breed had significant effects on chemical composition and meat quality parameters including color, water-holding capacity, dripping loss, thawing loss, and cooking loss values. From the point of view of consuming healthy meat, breeding A lambs could be suggested due to their low intramuscular fat and cholesterol contents. In addition dripping loss has an important effect on the quality of fresh meat and meat products; it was determined that the dripping loss value of A was lower than that of M, KR, or IW lambs.

## Data Availability

The data sets are available upon request from the corresponding author.
